# Comparison of mortality rates in patients with carbapenem-resistant *Enterobacterales* bacteremia according to carbapenemase production: a multicenter propensity-score matched study

**DOI:** 10.1038/s41598-023-51118-9

**Published:** 2024-01-05

**Authors:** Moon Seong Baek, Jong Ho Kim, Joung Ha Park, Tae Wan Kim, Hae In Jung, Young Suk Kwon

**Affiliations:** 1 Department of Internal Medicine, Chung-Ang University Hospital, Chung-Ang University College of Medicine, Seoul, Republic of Korea; 2 Department of Anesthesiology and Pain Medicine, College of Medicine, Chuncheon Sacred Heart Hospital, Hallym University, 77 Sakju-ro, Chuncheon, 24253 Republic of Korea; 3 Institute of New Frontier Research Team, Hallym University, Chuncheon, Republic of Korea; 4 Division of Infectious Diseases, Department of Internal Medicine, Gwangmyeong Hospital, Chung-Ang University, Gwangmyeong, Republic of Korea

**Keywords:** Diseases, Medical research

## Abstract

The spread of carbapenem-resistant *Enterobacterales* (CRE) poses a public health threat worldwide. We aimed to compare the mortality rates between the carbapenemase-producing (CP) and non-CP CRE bacteremia. We conducted a retrospective cohort study in patients with CRE bacteremia after propensity score (PS) matching. We performed a Kaplan–Meier curve analysis to identify the cumulative hazard for 30-day mortality. There were 318 patients with CRE between January 1, 2018, and December 31, 2022. There were 252 patients with CP-CRE and 66 with non-CP-RE, respectively. Before PS matching, the 30-day mortality rates were 40.9% in the non-CP-CRE group and 53.2% in the CP-CRE group (*p *= 0.097). In patients in the intensive care unit (ICU), the mortality rates were 49.0% in the non-CP-CRE group and 57.1% in the CP-CRE group (*p *= 0.340). After PS matching, the hazard ratio (HR) for mortality in the CP-CRE group was 1.49 (95% confidence interval [CI] 0.74–3.03), *p *= 0.266). In ICU patients, the HR of CP-CRE was 1.11 (95% CI 0.36–3.39, *p *= 0.860). The Kaplan–Meier curve for 30-day mortality showed no difference in cumulative hazard. After PS matching, there was no difference in 30-day mortality between patients with CP-CRE and non-CP-CRE bacteremia.

## Introduction

Carbapenems play a crucial role against gram-negative multi-drug resistant (MDR) pathogens. However, the spread of carbapenem-resistant *Enterobacterales* (CRE) poses a public health threat worldwide. CRE already have established endemicity in several regions, including North America, the Middle East, and North Africa^1^. Several risk factors, such as previous antibiotic exposure, a prolonged hospital stay, and immunosuppression, affect CRE acquisition^2,3^. Therefore, increasing use of empirical antibiotics, corticosteroids, or immunosuppressive medications during the coronavirus disease 2019 (COVID-19) pandemic could accelerate CRE in hospitals^4,5^. In particular, CRE is an important healthcare-associated infection pathogen, such as hospital-acquired pneumonia or bacteremia in the intensive care unit (ICU), and is associated with high mortality^3^.

Patients with CRE infection have a higher mortality rate than those with carbapenem-susceptible *Enterobacterales* infection^6^. Among CRE, *Enterobacterales* that inhibit carbapenem by producing carbapenemase are known as carbapenemase-producing (CP)-CRE^7^. CP-CRE is easily spread to other bacteria by transferring a plasmid^8^, and CP-CRE are the majority of CRE^9^. However, there is little research on the mortality rates of CP-CRE and non-CP-CRE infection. Tamma et al.^10^ suggested that CP-CRE had greater virulence and non-beta-lactam antibiotic resistance than non-CP-CRE. These differences lead to higher mortality rates in patients with CP-CRE bacteremia compared to those with non-CP-CRE bacteremia. However, Hovan et al.^11^ reported that patients with non-CP-CRE bacteremia had a 2.4 times higher risk of mortality than those with CP-CRE bacteremia. Owing to these contradictory results, we aimed to identify the mortality rate according to carbapenemase-producing bacteria in patients with CRE bacteremia.

## Results

### Patient characteristics

**Figure 1:**
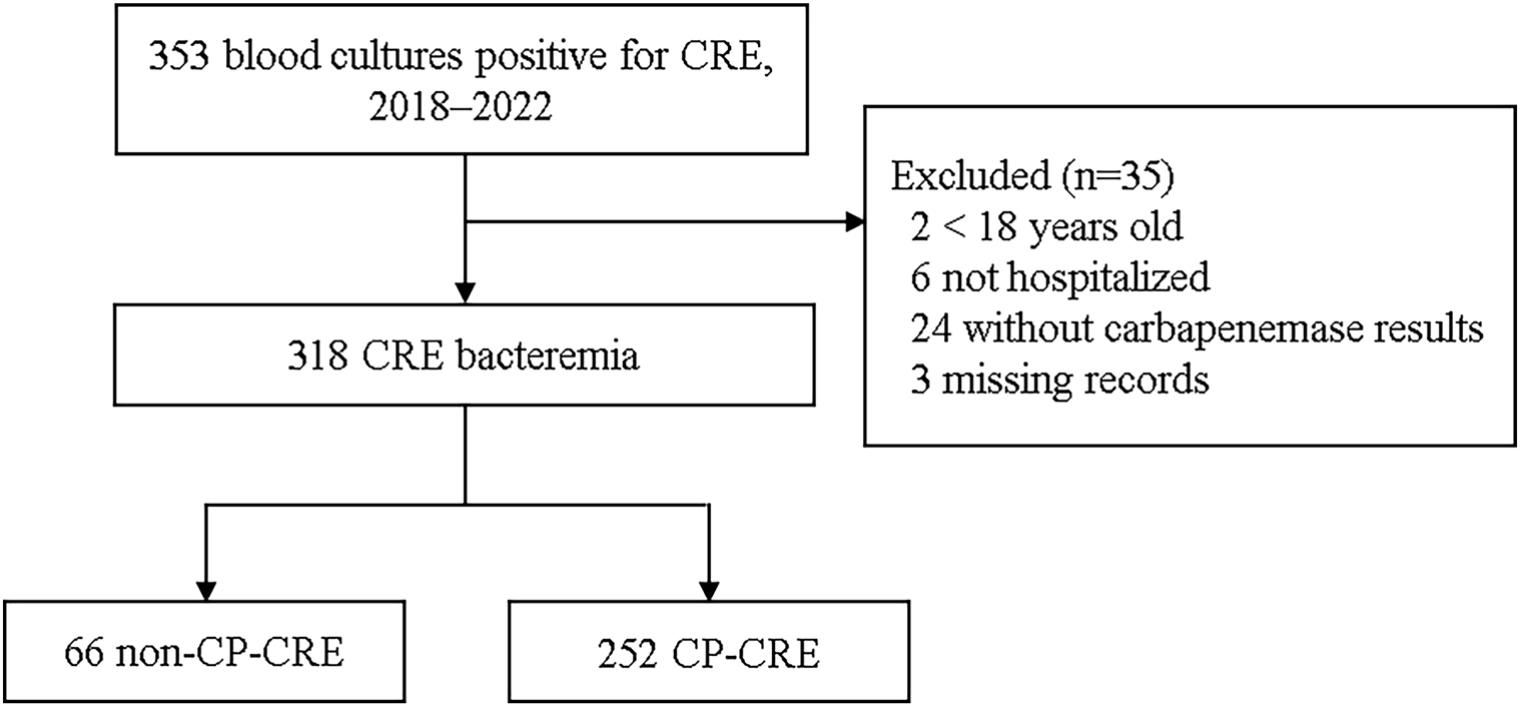
Flow diagram of the study population. CRE, Carbapenem-resistant *Enterobacterales*; and CP, carbapenemase-producing.

During the study period, we screened 353 CRE-positive patients on blood cultures. We excluded 35 subjects, including two < 18 years old, six not hospitalized, 24 missing carbapenemase results, and three missing records. Among the 318 subjects with CRE, 66 (21%) were non-CP-CRE and 252 (79%) were CP-CRE (Fig. 1). The participating centers and the number of CRE bacteremia are presented in Supplementary Table 1.

Table 1 shows the baseline characteristics of patients with CRE bacteremia. The average patient age was 67.4 (SD 15.7), and 61.6% of subjects were male. There was no difference in demographic data between non-CP-CRE and CP-CRE. The most common CRE identification location was the ICU; however, this was more common for those with CP-CRE (61.1% vs. 42.4%, *p *= 0.002). Hospital-acquired infection (HAI) was more frequent (83.3% vs. 68.2%, *p *= 0.010) and the APACHE II score was higher (16.7 ± 6.8 vs. 14.5 ± 7.0, *p *= 0.021) in those with CP-CRE compared to the non-CP-CRE group. The CP-CRE group had more feeding tubes than the non-CP-CRE group (65.5% vs. 36.4%, *p *< 0.001). The likely source of bacteremia is significantly different between two groups. The antibiotic treatment regimens for CRE bacteremia are described in Supplementary Table 2

**Table 1:** Baseline characteristics of patients with CRE bacteremia.

	Before PSM			After PSM			
	Non-CP-CRE (n = 66)	CP-CRE (n = 252)	*P* value	Non-CP-CRE (n = 66)	CP-CRE (n = 66)	*P *value	SMD
Age (yr)	70.2 ± 13.9	66.7 ± 16.1	0.103	70.2 ± 13.9	67.1 ± 16.4	0.234	0.208
Male (%)	37 (56.1)	159 (63.1)	0.366	37 (56.1)	35 (53.0)	0.525	0.111
Body mass index (kg/m^2^)	22.8 ± 3.8	22.7 ± 4.2	0.962	22.8 ± 3.8	22.8 ± 3.8	0.953	0.010
Location of blood culture (%)			0.002			0.562	0.188
Emergency room	17 (25.8)	26 (10.3)		17 (25.8)	12 (18.2)		
ICU	28 (42.4)	154 (61.1)		28 (42.4)	32 (48.5)		
General ward	21 (31.8)	72 (28.6)		21 (31.8)	22 (33.3)		
Hospital-acquired infection (%)	45 (68.2)	210 (83.3)	0.010	45 (68.2)	53 (80.3)	0.164	0.280
SOFA score	6.3 ± 3.9	7.0 ± 4.4	0.234	6.3 ± 3.92	5.6 ± 4.1	0.363	0.159
APACHE II score	14.5 ± 7.0	16.7 ± 6.8	0.021	14.5 ± 7.0	14.5 ± 6.9	0.990	0.002
Admission to blood culture, days	20.4 ± 31.2	36.0 ± 58.3	0.036	20.4 ± 31.2	27.9 ± 42.4	0.252	0.200
Blood culture to discharge, days	26.1 ± 32.8	34.4 ± 54.0	0.233	26.1 ± 32.8	33.6 ± 52.9	0.329	0.170
Prior organ transplantation (%)	2 (3.0)	13 (5.2)	0.689	2 (3.0)	3 (4.5)	1.000	0.079
Prior surgery (%)	19 (28.8)	102 (40.5)	0.110	19 (28.8)	23 (34.8)	0.575	0.130
Neutropenia (%)	7 (10.6)	17 (6.7)	0.427	7 (10.6)	7 (10.6)	1.000	< 0.001
Prior chemotherapy (%)	14 (21.2)	42 (16.7)	0.496	14 (21.2)	14 (21.2)	0.498	< 0.001
Prior steroid use (%)	9 (13.6)	39 (15.5)	0.858	9 (13.6)	9 (13.6)	0.790	< 0.001
Prior biologic or immunomodulators (%)	3 (4.5)	18 (7.1)	0.633	3 (4.5)	5 (7.6)	0.715	0.127
Central venous catheter (%)	43 (65.2)	196 (77.8)	0.051	43 (65.2)	43 (65.2)	1.000	< 0.001
Urethral catheter (%)	46 (69.7)	200 (79.4)	0.132	46 (69.7)	46 (69.7)	1.000	< 0.001
Feeding tube (%)	24 (36.4)	165 (65.5)	< 0.001	24 (36.4)	26 (39.4)	0.858	0.062
Percutaneous drain tube (%)	10 (15.2)	50 (19.8)	0.490	10 (15.2)	9 (13.6)	1.000	0.043
Septic shock (%)	17 (25.8)	52 (20.6)	0.465	17 (25.8)	9 (13.6)	0.126	0.308
ICU admission (%)	49 (74.2)	212 (84.1)	0.092	49 (74.2)	49 (74.2)	1.000	< 0.001
Likely source of bacteremia			< 0.001			0.804	0.307
Catheter-related infection	1 (1.5)	15 (6.0)		1 (1.5)	2 (3.0)		
Urinary tract infection	12 (18.2)	41 (16.3)		12 (18.2)	13 (19.7)		
Intra-abdominal infection	3 (4.5)	13 (5.2)		3 (4.5)	3 (4.5)		
Pneumonia	11 (16.7)	90 (35.7)		11 (16.7)	14 (21.2)		
Skin and soft tissue	1 (1.5)	18 (7.1)		1 (1.5)	3 (4.5)		
Primary bacteremia	37 (56.1)	68 (27.0)		37 (56.1)	29 (43.9)		
Others	1 (4.5)	7 (2.8)		1 (1.5)	2 (3.0)		
Treatment (%)							
Mechanical ventilation	30 (45.5)	162 (64.3)	0.008	30 (45.5)	35 (53.0)	0.486	0.152
CRRT	19 (28.8)	86 (34.1)	0.500	19 (28.8)	14 (21.2)	0.421	0.176
Vasopressors	36 (54.5)	148 (58.7)	0.636	36 (54.5)	29 (43.9)	0.296	0.213
Steroids	27 (40.9)	118 (46.8)	0.471	27 (40.9)	29 (43.9)	0.860	0.061
Opioids	6 (9.1)	52 (20.6)	0.047	6 (9.1)	8 (12.1)	0.777	0.099
Sedatives	13 (19.7)	89 (35.3)	0.023	13 (19.7)	10 (15.2)	0.646	0.120
Neuromuscular blockers	10 (15.2)	31 (12.3)	0.683	10 (15.2)	5 (7.6)	0.273	0.240
Type of CRE strain (%)			< 0.001			0.144	0.348
* Klebsiella pneumoniae*	25 (37.9)	213 (84.5)		25 (37.9)	36 (54.5)		
* Escherichia coli*	17 (25.8)	19 (7.5)		17 (25.8)	14 (21.2)		
Others	24 (36.4)	20 (7.9)		24 (36.4)	16 (24.2)		
Appropriate empirical treatment (%)	23 (34.8)	56 (22.2)	0.051	23 (34.8)	15 (22.7)	0.178	0.270
Appropriate definitive treatment (%)	37 (56.1)	102 (40.5)	0.033	37 (56.1)	36 (54.5)	1.000	0.030

Values expressed as mean ± SD or n (%).

*CRE* Carbapenem-resistant *Enterobacterales*, *PSM* Propensity-score matching, *CP* Carbapenemase-producing, *SMD* Standardized mean difference, *ICU* Intensive care unit, *SOFA* Sequential Organ Failure Assessment, *APACHE* Acute Physiologic Assessment and Chronic Health Evaluation, *CRRT* Continuous renal replacement therapy.

In the ICU, mechanical ventilation and sedatives were more commonly used in the CP-CRE group than the non-CP-CRE group (64.3% vs. 45.5%, *p *= 0.008; and 35.3% vs. 19.7%, *p *= 0.023). The rate of appropriate definitive treatment was lower in the CP-CRE group than in the non-CP-CRE group (40.5% vs. 56.1%, *p *= 0.033). Table 1 shows the standardized difference in means between the two groups after propensity-score (PS) matching.

### Characteristics of ICU patients

In ICU patients, the CP-CRE group had higher APACHE II scores and a higher incidence of HAI and feeding tube use than the non-CP-CRE group. Appropriate definitive treatment was lower in the CP-CRE group. Table 2 presents the post-PS matching results.

**Table 2:** Baseline characteristics of ICU patients with CRE bacteremia.

	Before PSM			After PSM			
	Non-CP-CRE (n = 40)	CP-CRE (n = 181)	*P* value	Non-CP-CRE (n = 40)	CP-CRE (n = 40)	*P* value	SMD
Age (yr)	69.3 ± 15.2	64.8 ± 16.8	0.122	69.3 ± 15.2	65.6 ± 17.3	0.313	0.227
Male (%)	25 (62.5)	116 (64.1)	0.994	25 (62.5)	21(52.5)	0.497	0.203
Body mass index (kg/m^2^)	22.7 ± 3.9	22.9 ± 4.4	0.801	22.7 ± 3.8	23.4 ± 5.2	0.521	0.144
Hospital-acquired infection (%)	30 (75.0)	161 (89.0)	0.038	30 (75.0)	36 (90.0)	0.141	0.403
SOFA score	7.4 ± 3.5	8.4 ± 4.2	0.143	7.4 ± 3.5	6.9 ± 3.9	0.588	0.122
APACHE II score	16.3 ± 6.7	18.6 ± 6.6	0.042	16.3 ± 6.7	16.7 ± 7.6	0.815	0.053
Admission to blood culture, days	23.6 ± 34.1	39.8 ± 57.6	0.089	23.6 ± 34.1	37.4 ± 37.5	0.090	0.384
Blood culture to discharge, days	33.3 ± 39.9	37.3 ± 57.3	0.676	33.3 ± 39.9	32.9 ± 44.8	0.966	0.009
Prior organ transplantation (%)	2 (5.0)	10 (5.5)	1.000	2 (5.0)	3 (7.5)	1.000	0.103
Prior surgery (%)	17 (42.5)	87 (48.1)	0.643	17 (42.5)	16 (40.0)	1.000	0.051
Neutropenia (%)	1 (2.5)	8 (4.4)	0.909	1 (2.5)	2 (5.0)	1.000	0.132
Prior chemotherapy (%)	5 (12.5)	20 (11.0)	1.000	5 (12.5)	6 (15.0)	1.000	0.073
Prior steroid use (%)	5 (12.5)	25 (13.8)	1.000	5 (12.5)	6 (15.0)	1.000	0.073
Prior biologic or immunomodulators (%)	2 (5.0)	12 (6.6)	0.981	2 (5.0)	3 (7.5)	1.000	0.103
Central venous catheter (%)	34 (85.0)	171 (94.5)	0.079	34 (85.0)	35 (87.5)	1.000	0.073
Urethral catheter (%)	36 (90.0)	166 (91.7)	0.970	36 (90.0)	36 (90.0)	1.000	< 0.001
Feeding tube (%)	23 (57.5)	146 (80.7)	0.004	23 (57.5)	24 (60.0)	1.000	0.051
Percutaneous drain tube (%)	9 (22.5)	43 (23.8)	1.000	9 (22.5)	5 (12.5)	0.377	0.265
Septic shock (%)	15 (37.5)	50 (27.6)	0.294	15 (37.5)	7 (17.5)	0.080	0.460
Likely source of bacteremia			0.042			0.818	0.390
Catheter-related infection	1 (2.5)	9 (5.0)		1 (2.5)	0 (0.0)		
Urinary tract infection	6 (15.0)	19 (10.5)		6 (15.0)	7 (17.5)		
Intra-abdominal infection	1 (2.5)	8 (4.4)		1 (2.5)	1 (2.5)		
Pneumonia	11 (27.5)	83 (45.9)		11 (27.5)	12 (30.0)		
Skin and soft tissue	1 (2.5)	16 (8.8)		1 (2.5)	2 (5.0)		
Primary bacteremia	19 (47.5)	41 (22.7)		19 (47.5)	15 (37.5)		
Others	1 (2.5)	5 (2.8)		1 (2.5)	3 (7.5)		
Treatment							
Mechanical ventilation (%)	27 (67.5)	149 (82.3)	0.059	27 (67.5)	29 (72.5)	0.807	0.109
CRRT (%)	18 (45.0)	80 (44.2)	1.000	18 (45.0)	16 (40.0)	0.821	0.101
Vasopressors (%)	28 (70.0)	126 (69.6)	1.000	28 (70.0)	24 (60.0)	0.482	0.211
Steroids (%)	18 (45.0)	87 (48.1)	0.860	18 (45.0)	19 (47.5)	1.000	0.050
Opioids (%)	6 (15.0)	49 (27.1)	0.163	6 (15.0)	11 (27.5)	0.274	0.309
Sedatives (%)	13 (32.5)	85 (47.0)	0.136	13 (32.5)	15 (37.5)	0.815	0.105
Neuromuscular blockers (%)	10 (25.0)	31 (17.1)	0.350	10 (25.0)	4 (10.0)	0.141	0.403
Type of CRE strain (%)			< 0.001			0.191	0.415
* Klebsiella pneumoniae*	17 (42.5)	161 (89.0)		17 (42.5)	25 (62.5)		
* Escherichia coli*	8 (20.0)	7 (3.9)		8 (20.0)	6 (15.0)		
Others	15 (37.5)	13 (7.2)		15 (37.5)	9 (22.5)		
Appropriate empirical treatment (%)	18 (45.0)	42 (23.2)	0.009	18 (45.0)	14 (35.0)	0.494	0.205
Appropriate definitive treatment (%)	26 (65.0)	71 (39.2)	0.005	26 (65.0)	23 (57.5)	0.646	0.154

Values expressed as mean ± SD or n (%).

*ICU* Intensive care unit, *CRE* Carbapenem-resistant *Enterobacterales*, *PSM* Propensity-score matching, *CP* Carbapenemase-producing, *SMD* Standardized mean difference, *SOFA* Sequential Organ Failure Assessment, *APACHE* Acute Physiologic Assessment and Chronic Health Evaluation, *CRRT* Continuous renal replacement therapy.

### CRE bacteremia prevalence and CRE strain distribution trends

**Figure 2:**
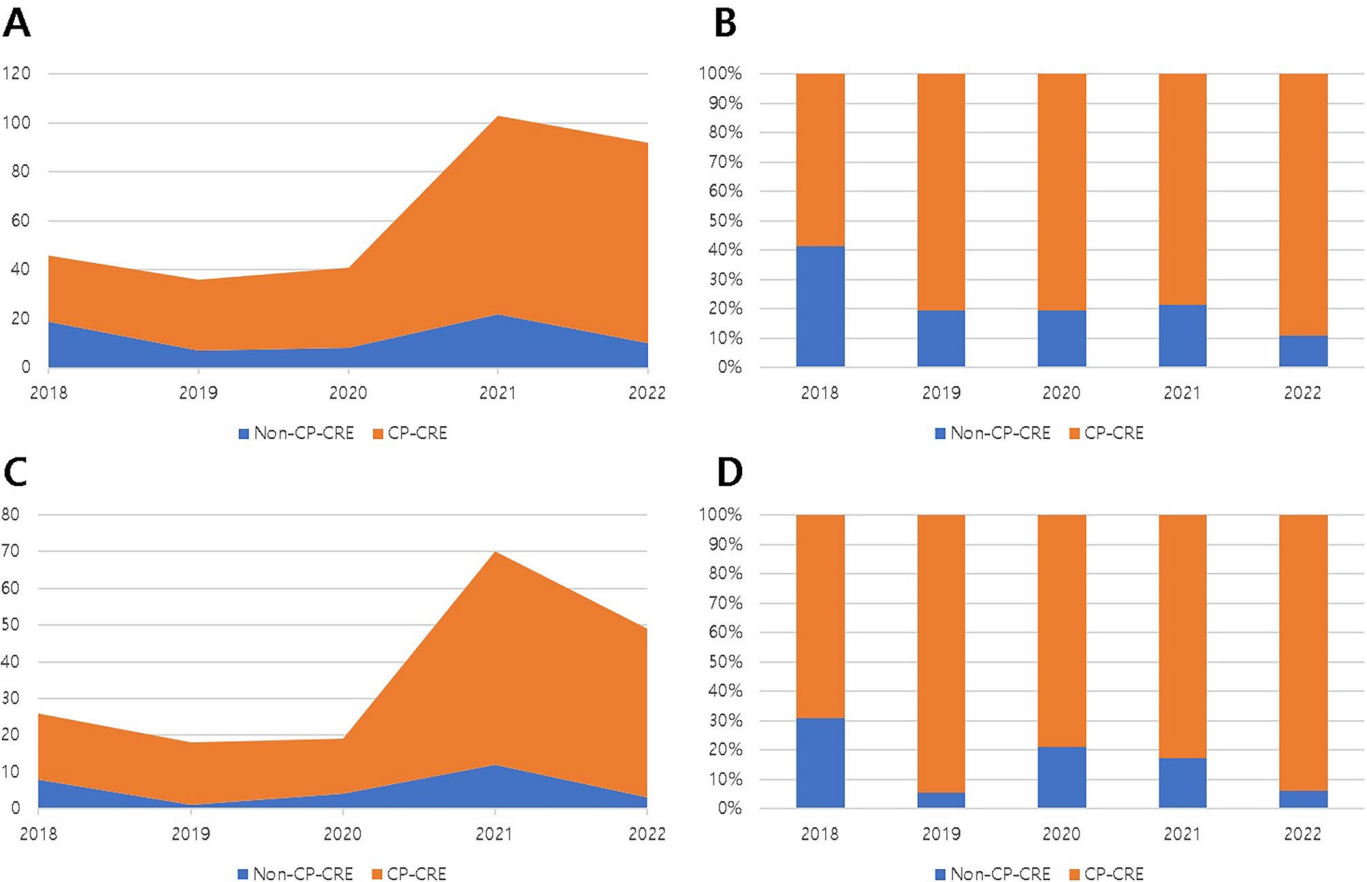
The prevalence of CRE bacteremia by year (2018–2022). (**A**) The incidence of CP-CRE versus non-CP-CRE in the overall cohort. (**B**) The percentage of CP-CRE versus non-CP-CRE in the overall cohort. (**C**) The incidence of CP-CRE versus non-CP-CRE among ICU patients. (**D**) The percentage of CP-CRE versus non-CP-CRE among ICU patients. CRE, carbapenem-resistant *Enterobacterales*; CP, carbapenemase-producing; ICU, intensive care unit.

The prevalence of CRE increased significantly since 2020 in the overall cohort and ICU patients (Fig. 2). The increase in CP-CRE led to an incremental increase in CRE bacteremia.

In the overall cohort, the most common CRE strains included *Klebsiella pneumoniae* at 74.8% (238 of 318 subjects) and *Escherichia coli* at 11.3% (36 of 318 subjects). The prevalence of *Klebsiella pneumoniae* was higher in the CP-CRE group than in the non-CP-CRE group (84.5% vs. 37.9%, respectively) (Table 1). Among 252 CP-CRE, there were 189 (75.0%) Klebsiella pneumoniae carbapenemase (KPC), 58 (23.0%) New Delhi metallo–β–lactamase (NDM), 9 (3.6%) oxacillinase (OXA)-48, one (0.4%) imipenemase (IMP). There were four co-existence cases (two KPC with OXA-48, one KPC with NDM, and one KPC combined with NDM and OXA-48). KPC was common in all CP-CRE strains: 74.6% (159/213) in *Klebsiella pneumoniae*, 73.7% (14/19) in *Escherichia coli*, and 80.0% (16/20) in other CP-CRE strains. In the ICU, the CRE strains and carbapenemases strains were similar to those of the overall cohort.

### 30-day mortality

**Figure 3:**
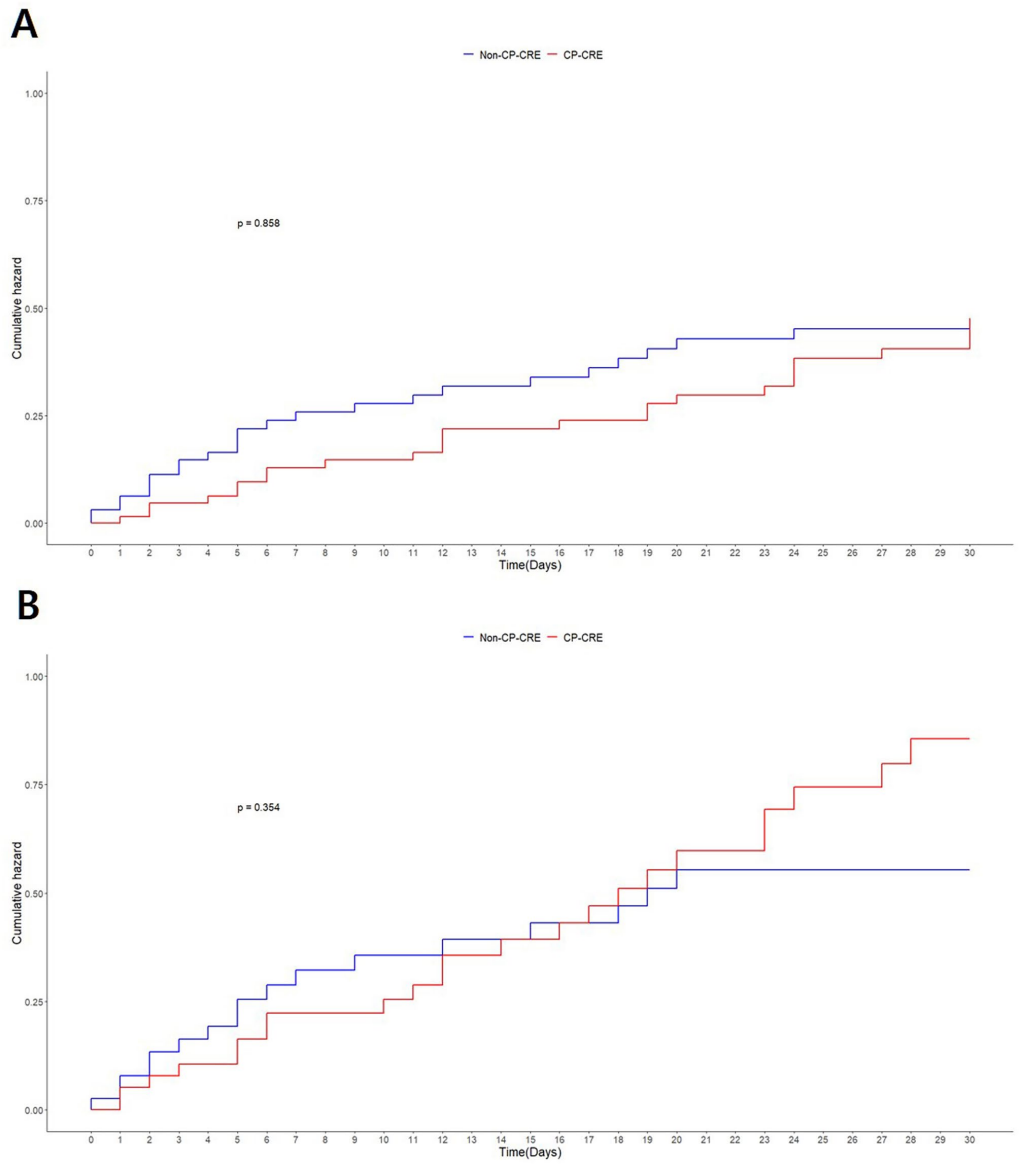
Kaplan–Meier curve analysis for 30-day mortality. (**A**) Overall cohort. (**B**) ICU patients. ICU, intensive care unit; CP, carbapenemase-producing; and CRE, carbapenem-resistant *Enterobacterales*.

The overall 30-day mortality rate was 50.6%. Before PS matching, the 30-day mortality rates were 40.9% in the non-CP-CRE group and 53.2% in the CP-CRE group (*p *= 0.097). In ICU patients, the rates were 49.0% in the non-CP-CRE group and 57.1% in the CP-CRE group (*p *= 0.340). Table 3 shows the Stratified Cox model analysis in the 1:1 PS-matched cohort. In multivariable model, the hazard ratio (HR) for mortality in the CP-CRE group was 1.49 (95% CI 0.74–3.03), *p *= 0.266). In ICU patients, the HR of CP-CRE was 1.11 (95% CI 0.36–3.39, *p *= 0.860). The Kaplan–Meier curve for 30-day mortality demonstrated no difference in cumulative hazard (Fig. 3).

**Table 3:** Stratified Cox model analysis in the propensity-score matching cohort.

30-day mortality in overall cohort	No. of patients	No. of Events	HR (95% CI)	*P* value
Univariate model				
CP-CRE	66	25	0.95 (0.54–1.67)	0.858
Non-CP-CRE	66	24		
Multivariable model^a^				
CP-CRE	66	25	1.49 (0.74–3.03)	0.266
Non-CP-CRE	66	24		
30-day mortality in ICU patients	No. of patients	No. of Events	HR (95% CI)	*P* value
Univariate model				
CP-CRE	40	23	1.33 (0.73–2.44)	0.354
Non-CP-CRE	40	17		
Multivariable model^b^				
CP-CRE	40	23	1.11 (0.36–3.39)	0.860
Non-CP-CRE	40	17		

*HR* Hazard ratio, *CI* Confidence interval, *CP* Carbapenemase-producing, *CRE* carbApenem-resistant *Enterobacterales*, *ICU* Intensive care unit.

^a^Adjusted for age, body mass index, location of the blood culture, hospital-acquired infection, SOFA score, days from admission to blood culture, days from blood culture to discharge, prior surgery, prior biologic or immunomodulators, septic shock, mechanical ventilation, CRRT, vasopressors, sedatives, neuromuscular blockers, type of CRE strain, appropriate empirical treatment, and likely source of bacteremia.

^b^Adjusted for age, sex, body mass index, hospital-acquired infection, SOFA score, days from admission to blood culture, prior organ transplantation, neutropenia, prior biologic or immunomodulators, percutaneous drain tube, septic shock, mechanical ventilation, CRRT, vasopressors, opioids, sedatives, neuromuscular blockers, type of CRE strain, appropriate empirical treatment, and likely source of bacteremia, and appropriate definitive treatment.

## Discussion

In this multicenter PS-matched study, we identified the clinical characteristics and mortality outcomes in patients with CP and non-CP CRE bacteremia. CP-CRE accounted for the majority of CRE, and CRE prevalence increased significantly since 2020. Compared to non-CP-CRE bacteremia, CP-CRE bacteremia was more common in the ICU and was associated with a higher APACH II score and use of mechanical ventilation. The overall 30-day mortality rate was high, but there was no significant difference in 30-day mortality rates in patients with CP and non-CP CRE bacteremia.

Several studies using data up to 2019 have compared CP-CRE bacteremia and non-CP-CRE bacteremia^10–13^ and reported that 14- or 30-day mortality rates ranged from 14 to 36.3%. Tamma et al. ^10^ described that the CP-CRE group had a 3.2 times higher 14-day mortality than the non-CP-CRE group (95% CI 1.06–9.61). However, the 30-day mortality rate was not statistically significant (adjusted odds ratio: 3.19, *p *= 0.050). Hovan et al.^11^ suggested a non-CP-CRE bacteremia had a higher 30-day mortality HR than CP-CRE bacteremia. They adjusted for renal replacement therapy and active antibiotic therapy between the two groups. However, as shown in our results, HAI, admission to blood culture interval, mechanical ventilation, and CRE strain type are confounding factors. Previous study populations were too small to determine the mortality difference between the two groups. Our study, which included a large number of patients with PS matching of potential confounding factors, found no difference in outcome between the two groups.

CP-CRE can be more virulent than non-CP-CRE. Jomehzadeh et al.^14^ found that CP-CRE had more virulence genes associated with siderophores, adhesins, and toxins. Furthermore, Tamma et al.^10^ asserted that CP-CRE can be more dangerous, as the carbapenems of the CP-CRE pathogens have superior hydrolyzing abilities compared non-CP-CRE. Several studies have shown that CP-CRE’s initial empirical antibiotic inappropriateness is higher than that of non-CP-CRE^10,12,13^. Therefore, initial empirical carbapenem treatment for CP-CRE bacteremia is more likely to fail than for non-CP-CRE bacteremia. In our study, CP-CRE was not an independent risk factor for mortality. Previous studies also demonstrated that CP-CRE and inappropriate empirical treatment were not risk factors for mortality^12,13^. Rather, hospital-acquired infection, higher APACHE II score, inappropriate definitive treatment, and mechanical ventilation contributed to death from CRE bacteremia. Although the virulence of CP-CRE is higher, its clinical significance is relatively small because the various factors mentioned above have a complex effect on death.

Our study found that the CP-CRE rate was increasing after the COVID-19 pandemic and ICU mortality of patients with CRE bacteremia was as high as 56%. Due to the widespread use of antimicrobial drugs during the COVID-19 pandemic, the increase in MDR organisms will accelerate^15^. According to a 2022 special report by the Centers for Disease Control and Prevention, the rate of CRE infections in hospitals increased by 35% in 2020 compared with 2019^16^. They suggested that patients who required an extended hospital stay during the pandemic may have contributed to the increase in CRE. In our study, patients with CP-CRE bacteremia had more devices, such as a central venous catheter or feeding tubes, and the ICU rate and HAI rate were high. Furthermore, most Korean ICUs contain multi-patient rooms and only one isolation unit is required for every 10 ICUs^17^. Therefore, the ICU may be vulnerable to cross-transmission of MDR organisms^18^. Therefore, expansion of ICU isolation facilities and infection control measures are needed to reduce MDR organisms, such as CRE, in the ICU ^3,19,20^.

This study has several limitations. First, due to the retrospective nature of the study some data could not be obtained such as carbapenem resistance mechanisms of non-CP-CRE or exact data for prior steroid use. Furthermore, although we collected data through the careful chart reviews, there is a possibility of misclassification of primary site of infection. Second, although we attempted PS matching using confounding variables between CP-CRE and non-CP-CRE bacteremia, potential confounders may remain. Third, this study population is limited to South Korea, therefore epidemiology may not be extrapolated to other countries or regions. Furthermore, this study did not include new beta-lactam antibiotics against MDR pathogens such as ceftalozane/tazobactam, ceftazidime-avibactam or cefiderol because these drugs were introduced recently in Korea. These can lead to lower appropriateness of definitive treatment, and may affect to generalibility of our results. Nonetheless, our multicenter study included a large study population and had consistent results in all subjects and ICU patients.

In conclusion, CP-CRE appears to be increasing after the COVID-19 pandemic. There was no difference in 30-day mortality between CP-CRE and non-CP-CRE bacteremia. However, the overall mortality of CRE bacteremia was 50.6%. In particular, CP-CRE bacteremia was closely associated with the ICU setting. More active infection control measures should be implemented to reduce CRE bacteremia.

## Methods

### Study design and patients

This multicentre study was conducted at Hallym University Medical Center, which includes 3100 patient beds in five university hospitals (Chuncheon Sacred Heart Hospital, Dongtan Sacred Heart Hospital, Kangnam Sacred Heart Hospital, Hangang Sacred Heart Hospital, and Hallym University Sacred Heart Hospital) across South Korea. The hospitals adopted the Clinical Data Warehouse (CDW) system to extract the electronic medical records. We retrospectively screened patients with a first CRE episode on blood culture through the CDW from January 1, 2018, to December 31, 2022. We excluded patients who were younger than 18 years of age, not hospitalized, with undetermined carbapenemase type, or missing data.

This study was approved by the Institutional Review Board of Chuncheon Sacred Hospital (No. 2022-08-010). All study procedures were performed according to relevant guidelines and regulations and followed the principles stipulated in the Declaration of Helsinki. Due to the retrospective nature of the study and after obtaining approval from the Institutional Review Board of Chuncheon Sacred Hospital, the requirement for informed consent from all participants was waived. This retrospective analysis was conducted on clinical data acquired post-treatment. This study followed the Strengthening the Reporting of Observational Studies in Epidemiology (STROBE) reporting guideline.

### Data collection

We collected demographic data, such as age, sex, and body mass index, the date and location of the blood culture (emergency room, ICU, or general ward), and the calculated Sequential Organ Failure Assessment and Acute Physiologic Assessment and Chronic Health Evaluation (APACHE) II scores at the onset of bacteremia. We collected data regarding organ transplantation (< three months), surgery (< three months), neutropenia (< three months), chemotherapy (< six months), biologic or immunomodulators (< one year), and steroid use (< one month) before bacteremia onset. We examined septic shock, ICU admission, and ICU treatments, including mechanical ventilation, renal replacement therapy, vasopressors, and steroid, opioid, sedative, and neuromuscular blocker use during hospitalization. We classified drainage catheters as central venous catheters, urethral catheters, feeding tubes, and percutaneous drain tubes, and CRE strains as *Klebsiella pneumoniae*, *Escherichia coli*, and others.

### Definitions

We defined the onset of bacteremia as the date of blood culture collection in which the CRE strain was first confirmed. We considered HAI as a positive blood culture obtained from a patient hospitalized for at least 48 hours^21^. We defined empirical antibiotic treatment as the antimicrobial agents given before obtaining the susceptibility results^22^. We defined appropriate empirical treatment as the use of antibiotics susceptible to the causative organism before the results of species identification and in vitro susceptibility. We defined appropriate definitive treatment as the administration of susceptible antibiotics based on in vitro susceptibility data^22^. The treatment outcome was 30-day mortality after the onset of bacteremia.

### Microbiology

Identification of microorganisms and susceptibility testing were performed at each clinical microbiology laboratory. The minimum inhibitory concentration (MIC)s of the Enterobacterales were determined using the Vitek 2 (bioMérieux Vitek, Hazelwood, MO, USA) or MicroScan system (Siemens, Sacramento, CA, USA). According to Clinical and Laboratory Standards Institute (CLSI) guideline^23^, carbapenem nonsusceptibility was determined by MIC > 1 mg/L for meropenem and imipenem or MIC > 0.5 mg/L for ertapenem. Among the CRE, the phenotypic detection of CP-CRE was conducted by modified Hodge test and carbapenemase inhibition test in-house. After the CRE isolates were sent to the Institute of Health and Environment, a carbapenemase gene polymerase chain reaction (PCR) test was performed to detect the presence of carbapenemase.

### Statistical analysis

We presented the continuous variables as the mean (standard deviation), and categorical variables as a number (percentage). We compared categorical variable data using the Chi-square test and continuous variables using the t-test.

We utilized the MatchIt package in R to conduct 1:1 Optimal Pair Matching^24^. This method minimizes the sum of absolute pairwise distances within the matched sample. Optimal pair matching offers several advantages, such as no requirement for a specified matching order and a reduced likelihood of large within-pair distances, compared to nearest-neighbor matching. In the overall cohort, the adjustment variables included the location of the blood culture, HAI, APACHE II score, admission to blood culture, feeding tube, mechanical ventilation, opioid use, sedative use, CRE strain, and appropriate definitive treatment. We used HAI, APACHE II score, feeding tube, CRE strains, appropriate empirical treatment, and appropriate definitive treatment for matching ICU patients.

We also performed a Kaplan–Meier curve analysis to identify the cumulative hazard for 30-day mortality. Then, we performed a stratified Cox proportional regression model to identify the cumulative hazard for 30-day mortality. Since this was a multicenter study, we considered a random-effect Cox proportional regression model to account for the center effect. We conducted both univariate and multivariate stratified Cox proportional regression models, and variables with a standardized mean difference greater than 10 were included in the multivariate model. We performed all statistical analyses using R version 4.2.2 (R Foundation for Statistical Computing). We considered *P* values less than 0.05 as statistically significant.

### Ethics approval and consent to participate

All procedures performed in this study were in accordance with the Declaration of Helsinki. The Institutional Review Board of Chuncheon Sacred Hospital (No. 2022-08-010) approved this study. The requirement for informed consent was waived due to the retrospective nature of the study.

### Supplementary Information

Supplementary Tables.

## Data Availability

The datasets used and analyzed in this study are available from the corresponding author upon reasonable request.
