# Supplementary material for: Comparison of mortality rates in patients with carbapenem-resistant Enterobacterales bacteremia according to carbapenemase production: a multicenter propensity-score matched study
Source: Sci Rep. 2024 Jan 5;14:597. doi: 10.1038/s41598-023-51118-9 (PMC10770160; doi:10.1038/s41598-023-51118-9)
Supplement: Supplementary file 1 — Supplementary Tables. [file 41598_2023_51118_MOESM1_ESM.pdf]

Comparison of mortality rates in patients with carbapenem-resistant *Enterobacterales* bacteremia according to carbapenemase production: a multicenter propensity-score matched study

Moon Seong Baek[1,*], Jong Ho Kim[2,3,*], Joung Ha Park[4], Tae Wan Kim[1], Hae In Jung[1], Young Suk Kwon[2,3]

[1]Department of Internal Medicine, Chung-Ang University Hospital, Chung-Ang University College of Medicine, Seoul, Korea

[2]Department of Anesthesiology and Pain Medicine, College of Medicine, Hallym University, Chuncheon Sacred Heart Hospital, Chuncheon, Republic of Korea

[3]Institute of New Frontier Research Team, Hallym University, Chuncheon, South Korea

[4]Division of Infectious Diseases, Department of Internal Medicine, Chung-Ang University Gwangmyeong Hospital, Gwangmyeong, South Korea

Supplementary Table 1. Participating centers and the number of CRE bacteremia

Participating centers	No. of CRE	No. of Non-CP-CRE	No. of CP-CRE
Chuncheon Sacred Heart Hospital	49	4	45
Dongtan Sacred Heart Hospital,	22	9	13
Kangnam Sacred Heart Hospital	62	27	35
Hangang Sacred Heart Hospital	44	10	34
Hallym University Sacred Heart Hospital	141	16	125

CRE, carbapenem-resistant *Enterobacterales*; and CP, carbapenemase-producing.

Supplementary Table 2. Antibiotic treatment regimens for CRE bacteremia

	Total (n = 318)	Non-CP-CRE (n = 66)	CP-CRE (n = 252)	P-value
Amikacin	84 (26.4)	9 (13.6)	75 (29.8)	0.008
Carbapenem	136 (42.8)	22 (33.3)	114 (45.2)	0.082
Colistin	127 (39.9)	12 (18.2)	115 (45.6)	<0.001
Piperacillin-tazobactam	42 (13.2)	9 (13.6)	33 (13.1)	0.908
Cefepime	20 (6.3)	5 (7.6)	15 (6.0)	0.578
Quinolone	24 (7.5)	9 (13.6)	15 (6.0)	0.062
Tigecycline	35 (11.0)	7 (10.6)	28 (11.1)	0.907
Gentamicin	18 (5.7)	2 (3.0)	16 (6.3)	0.384
Trimethoprim-Sulfamethoxazole	10 (3.1)	2 (3.0)	8 (3.2)	1.000
Ceftriaxone or cefotaxime	11 (3.5)	8 (12.1)	3 (1.2)	<0.001
Ceftazidime	3 (0.9)	1 (1.5)	2 (0.8)	0.504
Ampicillin-sulbactam	2 (0.6)	0 (0.0)	2 (0.8)	0.627
Combination therapy	172 (54.1)	19 (28.8)	153 (60.7)	<0.001
Combination with carbapenem regimen	93 (54.1)	8 (42.1)	85 (55.6)	0.267

CRE, carbapenem-resistant *Enterobacterales*; and CP, carbapenemase-producing.
